# Povidone-Iodine Attenuates Viral Replication in Ocular Cells: Implications for Ocular Transmission of RNA Viruses

**DOI:** 10.3390/biom11050753

**Published:** 2021-05-18

**Authors:** Sneha Singh, Onkar B. Sawant, Shahzad I. Mian, Ashok Kumar

**Affiliations:** 1 Department of Ophthalmology, Visual and Anatomical Sciences, Kresge Eye Institute, School of Medicine, Wayne State University, Detroit, MI 48201, USA; gq8860@wayne.edu; 2 Center for Vision and Eye Banking Research, Eversight, Cleveland, OH 44103, USA; osawant@eversightvision.org; 3 Kellogg Eye Center, Department of Ophthalmology and Visual Sciences, University of Michigan, Ann Arbor, MI 48105, USA; smian@med.umich.edu; 4 Department of Biochemistry, Microbiology, and Immunology, School of Medicine, Wayne State University, Detroit, MI 48201, USA

**Keywords:** povidone-iodine, SARS-CoV-2, inflammation, antiviral, Zika, Chikungunya

## Abstract

Several RNA viruses, including SARS-CoV-2, can infect or use the eye as an entry portal to cause ocular or systemic diseases. Povidone-Iodine (PVP-I) is routinely used during ocular surgeries and eye banking as a cost-effective disinfectant due to its broad-spectrum antimicrobial activity, including against viruses. However, whether PVP-I can exert antiviral activities in virus-infected cells remains elusive. In this study, using Zika (ZIKV) and Chikungunya (CHIKV) virus infection of human corneal and retinal pigment epithelial cells, we report antiviral mechanisms of PVP-I. Our data showed that PVP-I, even at the lowest concentration (0.01%), drastically reduced viral replication in corneal and retinal cells without causing cellular toxicity. Antiviral effects of PVP-I against ZIKV and CHIKV were mediated by direct viral inactivation, thus attenuating the ability of the virus to infect host cells. Moreover, one-minute PVP-I exposure of infected ocular cells drastically reduced viral replication and the production of infectious progeny virions. Furthermore, viral-induced (CHIKV) expression of inflammatory genes (*TNF-α*, *IL-6*, *IL-8*, and *IL1β*) were markedly reduced in PVP-I treated corneal epithelial cells. Together, our results demonstrate potent antiviral effects of PVP-I against ZIKV and CHIKV infection of ocular cells. Thus, a low dose of PVP-I can be used during tissue harvesting for corneal transplants to prevent potential transmission of RNA viruses via infected cells.

## 1. Introduction

RNA virus epidemics continue to threaten human health, with epidemics caused by Zika virus (ZIKV), Ebola virus (EBOV), Dengue virus (DENV), Chikungunya virus (CHIKV), and more recently the Severe Acute Respiratory Syndrome Coronavirus-2 (SARS-CoV-2), the causative agent of the COVID-19 pandemic [1,2,3,4]. We previously reported ocular complications due to flaviviruses [5], especially those caused by ZIKV [6,7,8]. Although the majority of ocular complications due to ZIKV infection have been shown to affect the retina, we reported ZIKV’s ability to infect primary corneal epithelial cells and trabecular meshwork cells, and to cause glaucoma [7,9]. Similarly, other clinical and experimental studies have reported the presence of ZIKV in tears, conjunctiva, and the ocular surface. Among the RNA viruses, CHIKV has also been reported to infect the human cornea and can be transmitted via the ocular route [3,10,11,12,13]. Together, these clinical and experimental pieces of evidence indicate RNA viruses can cause ocular surface complications. More recently, we showed the presence of SARS-CoV-2 in 13% of corneas from COVID-19-affected donors [14]. These findings underscore the possibility of potential transmission of SARS-CoV-2 via corneal transplant [15]. However, to minimize this risk, the Eye Bank Association of America (EBAA) recommends the use of povidone-iodine (PVP-I) to sterilize the donor eye prior to the harvesting of ocular tissues [16,17,18]. Although we were not able to definitively determine the effectiveness of PVP-I in our study because of the small sample size, we found that PVP-I-treated donor eyes were negative for SARS-CoV-2 RNA [14]. Given the increasing number of studies reporting the presence of SARS-CoV-2 in the ocular fluids, conjunctiva, and corneal and retinal tissues [14,19,20,21,22,23,24], diagnostic and preventative strategies are needed to prevent the ocular transmission of viruses.

PVP-I formulations have been widely used for more than 60 years because of their broad-spectrum antimicrobial activity and established safety profile [25,26]. PVP-I is on the WHO List of Essential Medicines, which identifies important medicines necessary for a functional healthcare system. PVP-I oral rinses and nasal sprays have also been advocated for by dental and head/neck surgeons to reduce the transmission of the SARS-CoV-2 virus [26,27,28]. Thus, PVP-I mouthwash is included in the WHO R&D Blueprint for experimental therapies against COVID-19. PVP-I has demonstrated in vitro activity against a range of viruses [29], including SARS-CoV-2 [30,31,32,33], the related SARS-CoV [34], and MERS-CoV [35]. While most of these studies have evaluated the direct antiviral activity of PVP-I, studies are lacking that assess the effect of PVP-I in viral-infected cells, especially those of ocular origin. 

In this study, we sought to determine the effect of PVP-I on viral replication in corneal and retinal cells infected with ZIKV or CHIKV, with these being a surrogate for single-stranded, positive-sense RNA viruses.

## 2. Materials and Methods

### 2.1. Cells and Culture Conditions

Human retinal pigment epithelial cells (ARPE-19 cell line, ATCC CRL-2302) were cultured using DMEM/F12 media (Gibco, ThermoFisher Scientific, Waltham, MA, USA) supplemented with 10% fetal bovine serum (Gibco, ThermoFisher Scientific, Waltham, MA, USA) [8,36]. Vero cells (ATCC CCL-81) were cultured in DMEM medium (Gibco, ThermoFisher Scientific, Waltham, MA, USA) supplemented with 10% FBS, while the human corneal epithelial cells (HUCL cell line) were maintained in a defined keratinocyte-serum-free medium (KBM-2, Lonza, Basel, Switzerland) in a humidified 5% CO_2_ incubator at 37 °C [9,37]. PVP-I reagent (ReadyPrep PVP-I 10% solution, Medline, Northfield, IL, USA) was used for the study. 

### 2.2. Virus Strains and Infection

Zika virus (ZIKV) Puerto Rican strain, PRVABC59 (NR-50240), and Chikungunya virus 181/25 clone, a live attenuated derivative of strain AF15561 (NR-13222) were obtained from BEI Resources, NIAID, NIH [7,8,9,38]. Both viruses were propagated in Vero cells and the viral titer was determined using the standard plaque assay method as described previously [38,39].

### 2.3. Plaque Assay

The viral titer was estimated by plaque assay, as mentioned in Singh et al., 2019 [38]. Briefly, the virus culture was serial diluted in serum-free media and allowed to adsorb on a monolayer of Vero cells for at least an hour. The culture supernatant was then aspirated, followed by the deposition of a monolayer of 2XDMEM with 4% Noble Agar (Sigma-Aldrich, St. Louis, MO, USA). The second overlay was added to the primary overlay on a subsequent day. The cells were fixed using 10% Trichloroacetic acid (TCA) for 10 min, followed by removal of the agar overlay and addition of Crystal violet stain. The plaques for CHIKV were counted at 2 days post-infection (dpi), while for ZIKV the plaques were counted at 6 dpi and expressed as PFU/mL.

### 2.4. Immunofluorescence Staining

The cells were cultured in 4-well chamber slides (Lab-Tek, Nunc) to 90% confluency, followed by infection with the virus (ZIKV or CHIKV). The cells were fixed using 4% paraformaldehyde (PFA) in 1X PBS at 4 °C, overnight. The cells were washed thrice with 1X PBS, followed by incubation in blocking and permeabilization buffer for an hour in a humidified chamber at room temperature. The cells were incubated with primary mouse monoclonal antibody 4G2 (1:100, Millipore, Billerica, MA, USA) for detection of ZIKV or rabbit anti-CHIKV E1 antibody (1:200, GeneTex, Irvine, CA, USA) overnight at 4 °C in a humidified chamber. The primary antibody was removed and washed thrice with 1X PBS, followed by incubation in anti-mouse/anti-rabbit Alexa 594 secondary antibody for one hour at 37 °C in a humidified chamber. The cells were washed thrice with 1X PBS and were mounted in Vectashield anti-fade mounting medium with DAPI (Vector Laboratories, Burlingame, CA, USA) to counterstain the nuclei in the cells. The cells were visualized and imaged using the Keyence fluorescence microscope BZ-X800 (Keyence, Itasca, IL, USA).

### 2.5. Real-Time PCR

Gene expression was quantified by the qPCR method previously described [8,38]. Total RNA was obtained using Trizol reagent (Life Technologies, Carlsbad, CA, USA) by following the manufacturer’s instructions. 2 μg of total RNA was reverse-transcribed using maxima cDNA synthesis kit (Thermo Fisher, Waltham, MA, USA) followed by qPCR using SYBr mix (Radiant™ SYBR Green Hi-ROX qPCR Kits, Alkali Scientific, Fort Lauderdale, FL, USA) and specific pairs of oligo primers previously used in our studies. Relative expression was calculated by the ΔΔCt method using *GAPDH* expression as a reference housekeeping gene. The primers used for amplifying the CHIKV viral RNA were CHIKV E1 Forward primer: 5′-AAGCTYCGCGTCCTTTACCAAG-3′, CHIKV E1 Reverse primer: 5′-CCAAATTGTCCYGGTCTTCCT-3′, and CHIKV E1 probe: FAM-CCAATGTCYTCMGCCTGGACACCTTT-TAMRA, as used by Kumar et al., 2021 [40]. The absolute quantification of CHIKV RNA from infected cells was performed as previously described using a standard curve method [8].

### 2.6. Cellular Toxicity Assay

Cell viability was determined using 3-(4,5-dimethylthiazol-2-yl)-2,5-diphenyltetrazolium bromide (MTT) assay (Invitrogen), as described in our previous study [38]. Briefly, cells were seeded in KBM-2 basal media in a 96-well plate overnight in a 37 °C incubator with 5% CO_2_. The cells were incubated with varying concentrations of PVP-I (0%, 0.01%, 0.05%, 0.1%, 0.5%, 1% and 2%) for 1 min, followed by three washes with 1X 

PBS and supplemented with fresh KBM-2 medium. The cells were incubated for 24 h at 37 °C, followed by the addition of 100 μL of MTT reagent (5 mg/mL in PBS) to each well for 4 h at 37 °C. The supernatant was then aspirated, followed by the addition of 100 μL of cell lysis buffer (20% SDS in 50% DMF) for an hour. The absorbance was measured using a microplate reader (Synergy multi-mode reader, BioTek, Winooski, VT, USA) and the cell viability was expressed as a percentage over control and calculated using the formula (mean OD of treated cells/mean OD of untreated control cells) × 100 and expressed as cell viability (%).

### 2.7. Statistical Analysis

The experiments were performed in biological triplicates for reproducibility and statistical significance. The statistical analysis was performed using GraphPad Prism 8 software with one-way ANOVA analysis. 

## 3. Results

### 3.1. PVP-I Directly Inactivates Enveloped RNA Viruses

**Figure 1:**
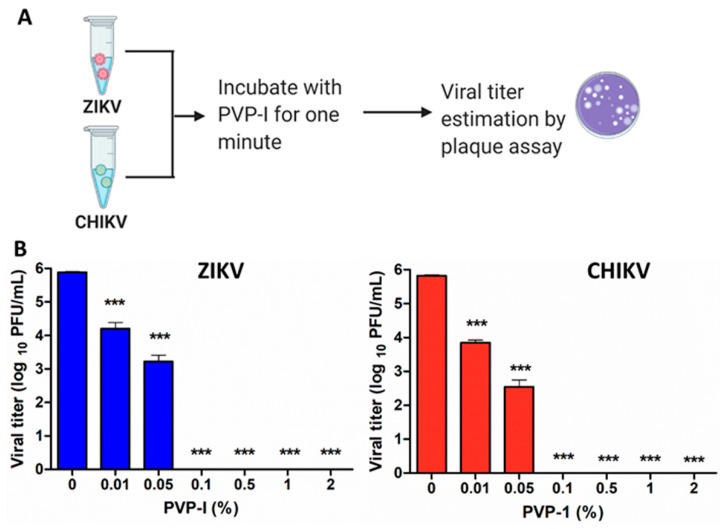
Direct inactivation of enveloped RNA viruses by PVP-I. (**A**) Schematic representation of the method used to assess the virucidal activity of PVP-I. (**B**) Zika virus (ZIKV) and Chikungunya virus (CHIKV) were incubated with indicated concentrations of PVP-I for one minute followed by plaque assay on Vero cells. The viral titer was expressed as log_10_ PFU/mL. One-way ANOVA with Dunnett’s test was used for statistical analysis wherein, *** *p* < 0.001.

PVP-I is known to exhibit virucidal activity against both RNA- and DNA-enveloped as well as non-enveloped viruses [26,29]. Therefore, we first assessed whether PVP-I directly inactivates the Zika virus and Chikungunya virus, which are both capable of causing ocular anomalies [3,5,7,8,9,41,42]. Both viruses (1 × 10^6^ PFU) were exposed to a gradient of PVP-I (0%, 0.01%, 0.05%, 0.1%, 0.5%, 1% and 2%) for one minute, followed by plaque assay on Vero cells (Figure 1A).

Our data showed a dose-dependent reduction in viral titers starting with 0.01% PVP-I treatment, and reaching 100% (6 log titer reduction) inactivation by 0.1% and higher concentrations of PVP-I (Figure 1B). The reduction in viral titer was very similar for both ZIKV and CHIKV. As expected, there was no evident reduction in the plaque size of untreated virus cultures. 

### 3.2. PVP-I Treatment Attenuates Viral Replication in Human Retinal Pigment Epithelial Cells

**Figure 2:**
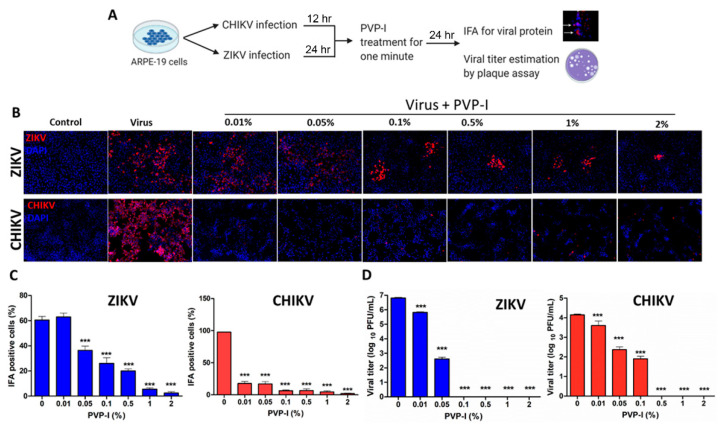
PVP-I exposure attenuates viral replication and production of viral progeny in retinal pigment epithelial cells. Schematic representation of the experimental design (**A**). ARPE-19 cells were infected with ZIKV and CHIKV for 24 h and 12 h, respectively. Infected cells were treated with indicated concentrations of PVP-I for one minute and extensively rinsed to remove residual PVP-I, and cultured for another 24 h in fresh media. Viral replication was assessed by immunofluorescent detection of viral antigens (red) using anti-Flavivirus 4G2 (ZIKV) and anti-CHKV E1 antibodies in fixed cells. The cell nuclei were counterstained using DAPI (blue). The images were captured at 20X magnification using the Keyence BZ-X800 series microscope (**B**). The cells expressing viral protein were counted and presented as IFA-positive cells (%) relative to the total number of cells from four independent fields (**C**). The culture supernatant was used to perform plaque assay on the Vero cell monolayer and the viral titer was expressed as log_10_ PFU/mL (**D**). One-way ANOVA with Dunnett’s test was used for statistical analysis, wherein *** *p* < 0.001.

We previously showed that ZIKV, to gain entry into the eye, can readily infect the RPE and retinal endothelial cells constituting the outer and inner blood-retinal barriers [5,8,38]. First, we assessed whether PVP-I causes toxicity in RPE and corneal epithelial cells by exposing cells with varying concentrations (0%, 0.01%, 0.05%, 0.1%, 0.5%, 1% and 2%) of PVP-I and found no significant cell death, as measured by MTT assay up to 0.5% PVP-I concentration (Supplementary Figure S1). Next, we sought to determine the effect of PVP-I on virus-infected RPE cells. ARPE-19 cells were infected with ZIKV or CHIKV for 24 and 12 h, respectively, to allow virus replication. Afterwards, these cells were exposed to a gradient of PVP-I for one minute and thoroughly rinsed with sterile PBS to remove residual PVP-I, and then incubated in normal growth media for another 24 h. The cells were processed for immunofluorescence detection of viral antigens, whereas the culture supernatant was used for viral progeny estimation (Figure 2A).

As expected, ARPE-19 cells were permissive to ZIKV, and similarly, CHIKV was also found to infect these cells. Interestingly, after the infectious dose of MOI 1, ZIKV caused infection in 60% of cells, whereas the infectivity rate with CHIKV was close to 100%, as evidenced by the increased fluorescence of viral antigens in untreated cells. However, brief exposure of infected cells to PVP-I significantly reduced viral replication, as indicated by reduced immunostaining of ZIKV or CHIKV antigens (Figure 2B). Quantification of the number of infected cells revealed that even the lowest concentration of PVP-I (i.e., 0.01%) effectively reduced replication of CHIKV, while 0.05% PVP-I significantly reduced the replication of ZIKV in the cells (Figure 2C). This was further confirmed by plaque assay, which showed a significant reduction in viral titers of cells exposed to 0.01% or higher concentration of PVP-I, with some variation in ZIKV compared to CHIKV. However, at ≥0.1% concentration of PVP-I, viral reduction was almost 100% (Figure 2D).

### 3.3. PVP-I Exposure Attenuated CHIKV Replication in Corneal Epithelial Cells

**Figure 3:**
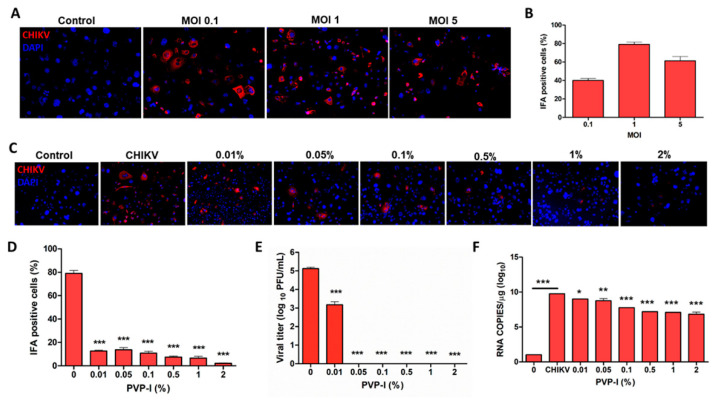
Corneal epithelial cells are permissive to CHIKV infection and PVP-I exposure attenuates viral replication. Human corneal epithelial cells (HUCL cell line) seeded on lab-tek chamber slides were infected with CHIKV at different MOIs (0.1, 1 and 5) for 12 h followed by immunofluorescence staining (**A**) and quantitation of viral antigen-positive cells (**B**). In another experiment 12 h CHIKV-infected (MOI 0.1) HUCL cells were incubated with varying concentrations of PVP-I for one minute. After PVP-I exposure, cells were rinsed and allowed to grow for 24 h in a fresh medium. Viral replication was determined by immunofluorescence staining (**C**) and quantification of CHIKV antigen-positive cells (**D**)**,** plaque assay (**E**), and assessment of viral RNA copy number by qPCR (**F**). One-way ANOVA Dunnett’s test was used for statistical analysis wherein, *** *p* < 0.001; ** *p* < 0.01, * *p* < 0.05.

Because the ocular surface is a gateway for viral entry, including SARS-CoV-2 [14,43], we sought to determine whether PVP-I can exert antiviral effects in the cornea. Moreover, according to the Eye Bank Association of America (EBAA) Medical Standards E1.100, PVP-I is routinely used to sterile the ocular surface during surgeries and while harvesting ocular tissue for transplant [14,18,32]. Previously, we reported that primary, but not immortalized corneal epithelial cells, are susceptible to ZIKV infection [9,14]. Therefore, we decided to use CHIKV infection of the corneal epithelium to evaluate the effect of PVP-I. First, we infected cells at different MOIs to determine their susceptibility to CHIKV. Our data showed that HUCL cells were permissive to CHIKV infection ranging from MOI 0.1 to MOI 5, with cytopathic effects at MOI 5 (Figure 3A,B). Therefore, we chose MOI 0.1 to infect HUCL cells in all of our experiments. 

As for RPE cells, the antiviral effects of PVP-I were assessed by briefly exposing CHIKV-infected HUCL cells to PVP-I. To this end, our data showed that ≥0.01% of PVP-I significantly reduced CHIKV replication, as evidenced by a reduction in CHIKV antigen-positive cells (Figure 3C,D). The antiviral effect of PVP-I was confirmed by plaque assay, which showed a significant reduction in progeny virion production in the cells exposed to ≥0.01% PVP-I (Figure 3E). Moreover, the viral copy number is also reduced in PVP-I-treated cells (Figure 3F). Together, these results indicate antiviral effects of PVP-I on CHIKV-infected corneal epithelial cells.

### 3.4. PVP-I Attenuates CHIKV-Induced Inflammatory Response in Corneal Epithelial Cells

**Figure 4:**
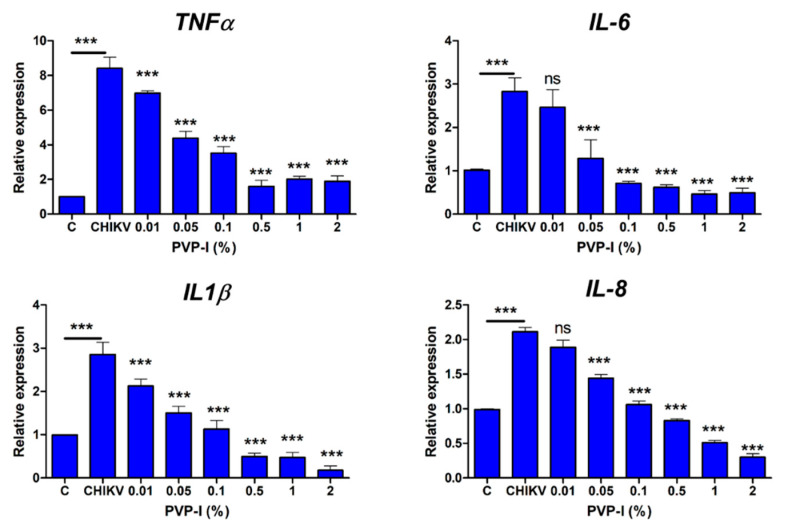
PVP-I treatment reduces CHIKV-induced inflammatory response in corneal epithelial cells. Human corneal epithelial cells (HUCL cell line) were infected at MOI 0.1 for 12 h and exposed to a gradient of PVP-I for one minute. The cells were rinsed and cultured in fresh media for an additional 24 h, followed by isolation of total RNA and cDNA synthesis. The expression of indicated inflammatory genes was assessed by qPCR and the fold change was calculated using the ΔΔCt method. The data are presented as relative expressions of genes in CHIKV-infected and PVP-I-untreated versus PVP-I-treated cells using *GAPDH* as the housekeeping gene. One-way ANOVA Bonferroni test was used for the statistical analysis wherein, *** *p* < 0.001; ns, not significant.

In response to infection, host cells produce inflammatory cytokines and chemokines to activate the innate immune system. Since PVP-I exposure reduced viral replication in CHIKV-infected HUCL cells, we next assessed the effect of PVP-I on the inflammatory response. The HUCL cells were infected with CHIKV 181/25 strain at MOI 0.1 and allowed to grow for 12 h, followed by treatment with a gradient of PVP-I (1 min). The cells were extensively rinsed with sterile PBS to remove any residual PVP-I and cultured in a fresh medium for 24 h at 37 °C. The cells were harvested for qPCR analysis of inflammatory genes. As anticipated, CHIKV induced the expression of inflammatory genes *IL1β*, *IL-6*, *IL-8* and *TNFα* in HUCL cells, and PVP-I exposure attenuated this response in a dose-dependent manner (Figure 4).

Our data showed a significant reduction in the expression of *TNFα* and *IL1β* at ≥0.01% PVP-I and at ≥0.05% PVP-I for *IL-6* and *IL-8*. 

## 4. Discussion

Pre-treatment with PVP-I has been shown to be effective in inactivating a variety of enveloped and non-enveloped viruses. The free iodine in PVP-I is mainly responsible for its antiviral action as it oxidizes crucial pathogen structures—amino acids, nucleic acid, and membrane components [44]. Here, we investigated the mechanisms underlying PVP-I antiviral activity in ZIKV- and CHIKV-infected corneal and retinal cells. We demonstrated that in addition to the direct inactivation of viruses, PVP-I exerted its antiviral activity in virus-infected cells, resulting in the reduced production of viable viral progeny. Together, our study suggests that a low dose of PVP-I could be an effective agent in preventing the potential transmission of viruses via ocular tissue transplants. 

PVP-I has been shown to have an in vitro efficacy of 0.23% for SARS CoV and MERS CoV, while it acts specifically against SARS-CoV-2 at concentrations as low as 0.5% and with contact time as low as 15 s [45,46]. There are multiple studies on the effect of PVP-I and its use as an oral rinse, antiseptic solution, intranasal rinse, etc. [26,27,29,35,46,47]. No studies have shown the effect of PVP-I on the emerging RNA virus-infected ocular cells in vitro or on ocular tissues to date. Therefore, we aimed to study the mechanism of action of PVP-I on RNA-enveloped viruses using our in vitro cell culture model of human corneal (HUCL cell line) and retinal pigment epithelial cells (ARPE-19 cell line). 

We previously reported that ARPE-19 and, to a lesser extent, HUCL cells were permissive to ZIKV infection [6,8,9], and that ZIKV does not cause significant cytopathic effects in these cells. In contrast, here, we observed that both ARPE-19 and HUCL are highly permissive to CHIKV infection and displayed early (<12 h) cytopathic effects, i.e., cell death and synapse formation. To assess the antiviral role of PVP-I against the enveloped RNA viruses in ocular cells, we infected ARPE-19 cells optimally with CHIKV and ZIKV, followed by one-minute exposure of the infected cells to PVP-I. Our findings indicate that there was a significant reduction in the viral replication detected by immunofluorescence staining of viral proteins. These findings were confirmed by a significant decrease in the production of progeny virions after short-term PVP-I treatment. Our results corroborate with the antiviral findings of [48]’s study, which describes PVP-I acting on human and avian influenza viruses in MDCK cells by blocking viral attachment to the host cell receptors and inhibiting viral release from infected cells. However, we found that PVP-I was more effective in inhibiting the replication of ZIKV compared to CHIKV. The differential effect of PVP-I antiviral activity has been previously studied, where a lower dose was found to be effective against measles, mumps, herpes, HIV, influenza, and rota-viruses, while rubella, polio-, adeno- and rhino-viruses were only sensitive to higher doses [49]. The safety of PVP-I has been proven in various clinical studies, including ophthalmology, otology, rhinology, and dermatology studies [32]. Our data showed that PVP-I did not have any significant cytopathic effect on the ARPE-19 cells and HUCL cells, even concentrations as high as 1% PVP-I (*v*/*v*), which is corroborated by similar cytotoxicity studies on MDCK, human fibroblast cells (Supplementary Figure S1). To our knowledge, our study is the first to demonstrate the protective effects of PVP-I against RNA viruses in ocular cells. Moreover, the higher infectivity of corneal epithelial cells to CHIKV can be used as a model to gauge antiviral effects of potential drugs and molecules.

The infection of HUCL cells with CHIKV elicited an inflammatory immune response at the transcript level, which was significantly reduced after PVP-I treatment on the cells in a dose-dependent manner. Although a pre-clinical and efficacy study has shown anti-inflammatory effects of PVP-I on wound healing, [24] where PVP-I and iodine favor wound healing by destroying microbial pathogen factors and tissue-destructive enzymes and cytokines, thus facilitating the wound healing process [50,51,52,53]. However, studies that elucidate the role of PVP-I in modulating host innate inflammatory response during viral infection are currently limited. One of the unique aspects of our study is to demonstrate that PVP-I treatment drastically reduced the production of inflammatory cytokines in ZIKV- and CHIKV-infected ocular cells. These inflammatory mediators provide host defense by the recruitment of innate immune cells to the site of infection and by alerting neighboring uninfected cells [38]. However, excessive and uncontrolled inflammation can cause collateral damage to the tissue. The observed anti-inflammatory effects of PVP-I observed in this study are likely due to the reduced replication of the viruses in corneal and retinal cells. 

In our previous study, we treated the COVID-19-affected donor eyes with and without 5% PVP-I for 5 min, and performed qPCR detection for SARS-CoV-2 on the anterior and posterior corneal tissue. Our results were inconclusive in demonstrating the effectiveness of betadine on SARS-CoV2 inactivation in donor tissues [14]. Therefore, further studies are required on a larger number of corneal donor tissues from individuals with COVID-19, or those infected with ZIKV or CHIKV, followed by treatment with PVP-I to study its antiviral effect on human corneal tissues. Using ZIKV and CHIKV, as surrogate-enveloped positive-strand RNA viruses, our study confirms the antiviral role of PVP-I on corneal epithelial cells. Therefore, we propose that PVP-I is likely to exert similar effects in SARS-CoV-2-infected ocular cells due to the similarities among enveloped-viruses. We acknowledge that the effective and safe concentrations of PVP-I used in vitro as compared to in vivo are different due to the complexity of the tissue layers and their tolerance. In tissues, a concentration of 0.25% was used for SARS-CoV-2 as a nasal spray and a concentration of 0.6% was used as a mouthwash. In contrast, eye banks and most ocular surgeries use 5% PVP-I to disinfect the eye. This is consistent with current EBAA Medical Standards E1.100, which recommend utilizing PVP-I solution prior to recovery of ocular tissues. 

In summary, our study demonstrates that PVP-I exerts its antiviral and anti-inflammatory effects on enveloped positive single-stranded RNA viruses by (a) direct contact inhibition of the virus, (b) attenuation of viral replication and the production of progeny virion, and (c) reducing the viral-induced inflammatory response. Thus, topical application of PVP-I can be explored to prevent the ocular transmission of RNA viruses, including SARS-CoV-2.

## Data Availability

Data is contained within the article or supplementary material.

## Supplementary Materials

The following are available online at https://www.mdpi.com/article/10.3390/biom11050753/s1, Figure S1: Assessment of cytotoxic effects of PVP-I on cultured ocular cells.
