# Supplementary material for: Povidone-Iodine Attenuates Viral Replication in Ocular Cells: Implications for Ocular Transmission of RNA Viruses
Source: Biomolecules. 2021 May 18;11(5):753. doi: 10.3390/biom11050753 (PMC8157382; doi:10.3390/biom11050753)
Supplement: Supplementary file 1 [file biomolecules-11-00753-s001.zip › biomolecules-1196192-supplementary.pdf]

biomolecules

MDPI

Supplementary materials

Povidone Iodine Attenuates Viral Replication in Ocular Cells: Implications for Ocular Transmission of RNA Viruses

Sneha Singh [1], Onkar B. Sawant [2], Shahzad I. Mian [3] and Ashok Kumar [1,4,*]

[1] Department of Ophthalmology, Visual and Anatomical Sciences, Kresge Eye Institute, Wayne State University School of Medicine, Detroit, MI, USA
[2] Center for Vision and Eye Banking Research, Eversight, Cleveland, OH, USA
[3] Kelloge Eye Center, Department of Ophthalmology and Visual Sciences, University of Michigan, Ann Arbor, MI, USA
[4] Department of Biochemistry, Microbiology, and Immunology, Wayne State University School of Medicine, Detroit, MI, USA
* Correspondence: akuma@med.wayne.edu

Citation: Singh, S.; Sawant, O.B.; Mian, S.I.; Kumar, A. Povidone Iodine Attenuates Viral Replication in Ocular Cells: Implications for Ocular Transmission of RNA Viruses. *Biomolecules* **2021**, *11*, 753. https://doi.org/10.3390/biom11050753

Academic Editor: Hemant Khanna

Received: 08 April 2021
Accepted: 12 May 2021
Published: 18 May 2021

Publisher's Note: MDPI stays neutral with regard to jurisdictional claims in published maps and institutional affiliations.

Copyright: © 2021 by the authors. Licensee MDPI, Basel, Switzerland. This article is an open access article distributed under the terms and conditions of the Creative Commons Attribution (CC BY) license (http://creativecommons.org/licenses/by/4.0/).

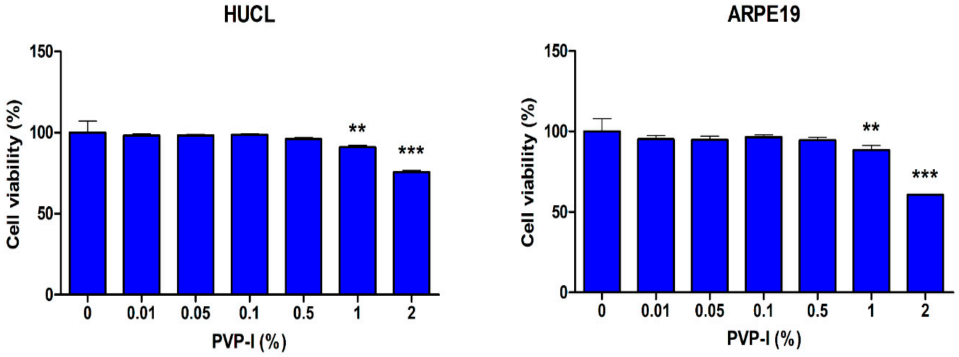

Figure S1: Assessment of cytotoxic effects of PVP-I on cultured ocular cells. HUCL and ARPE-19 cells were seeded in a 96-well plate followed by exposure to PVP-I at different concentrations for a minute. The cells were washed with 1X PBS (4 times) followed by culture in fresh medium for 24 hours. MTT assay was performed on the cells and expressed and cell viability (%) compared to control untreated cells. .
